# Diagnosis of Vertical Root Fractures in Endodontically Treated Teeth by Cone-Beam Computed Tomography

**DOI:** 10.3390/jimaging8030051

**Published:** 2022-02-23

**Authors:** Fumi Mizuhashi, Yuko Watarai, Ichiro Ogura

**Affiliations:** 1 Department of Removable Prosthodontics, The Nippon Dental University School of Life Dentistry at Niigata, Niigata 951-8580, Japan; watarai@ngt.ndu.ac.jp; 2 Department of Oral and Maxillofacial Radiology, The Nippon Dental University School of Life Dentistry at Niigata, Niigata 951-8580, Japan; ogura@ngt.ndu.ac.jp

**Keywords:** root fracture, diagnosis, vertical root fracture, cone-beam computed tomography, endodontically treated teeth

## Abstract

The purpose of this study was to investigate the characteristics and the detection ability of vertical root fractures in endodontically treated teeth by intraoral radiography and cone-beam computed tomography (CBCT). CBCT images of 50 patients with root fractures in endodontically treated teeth were reviewed, and 36 vertical root fractures were taken in this study. The cause of fracture, core construction, kind of teeth, and fracture direction (bucco-lingual and mesio-distal fractures) were investigated. Detection ability of vertical root fractures by intraoral radiography and CBCT was also examined. Statistical analyses concerning the characteristics were performed by χ^2^ test, and the detection ability was analyzed by cross-tabulation. All of the fractured teeth were nontraumatized teeth. The vertical root fracture occurrence was not differed by core construction. The vertical root fracture number was largest at the premolar teeth (*p* = 0.005), and the number of the bucco-lingual fracture was larger than the mesio-distal fracture (*p* = 0.046). Vertical root fractures were detectable using CBCT, while undetectable by intraoral radiography (*p* < 0.001). Vertical root fractures occurred easily in premolar teeth with bucco-lingual direction, and CBCT is an adequate radiographic method to diagnose vertical root fracture.

## 1. Introduction

In recent years, the prevention of caries or periodontal disease was developed, and causes of tooth loss changed from caries or periodontal disease to root fractures [1]. The causes of root fractures are trauma, occlusion, and so on. Root fractures easily occur in endodontically treated teeth compared to that of nonendodontic-treated teeth [2]. The characteristics of the root canal anatomy that would influence the occurrence of root fracture differ among the kind of teeth. For example, incisor is single canal for the most part [3]. In the maxillary premolar, the most prevalent root canals were the two canals, while most mandibular premolars have one canal [4]. Most of the maxillary first molar has three separate roots [5], and the mandibular first molar mesial roots have two canals [6]. The number of vertical root fractures is reported to be greater than that of horizontal root fracture [7]. Endodontic retreatment had the highest risk of vertical root fracture within 1–8 years [7]. Tooth loss by the vertical root fracture increased, and the percentage of extractions due to vertical root fracture is 31.7% [8]. It is important to diagnosis the vertical root fracture timely to avoid progressive alveolar bone loss [9]. Additionally, diagnosing the vertical root fracture in a timely fashion enables to treat the root-fractured tooth and avoid extraction.

On the root-fractured tooth, the specific clinical signs and symptoms are often unclear, and it is difficult to recognize the fracture before the tooth is extracted [10]. Among the radiographic methods to diagnose root fracture, periapical radiography is the primary means. However, the detection ability of periapical radiography is limited because it produces a 2D image with anatomical noise or geometric distortion [11,12].

Cone-beam computed tomography (CBCT) provides a 3D image that largely eliminates anatomical noise and suffers minimal geometric distortion. CBCT has higher accuracy compared to that of periapical radiography for the detection of vertical root fracture [13,14]. However, the accurate detection of vertical root fracture using CBCT is still under discussion [10,15,16]. One report mentioned that the fracture orientation of the vertical root fractures in teeth with intracanal metal post plays a role in the detection accuracy of CBCT [17]. Other reports showed that radiopaque materials used as root canal filling, such as gutta-percha, cause artifacts in CBCT images by causing beam hardening, and the diagnostic quality is therefore reduced [18,19,20]. On the other hand, CBCT successfully detects root fracture in both endodontically and nonendodontically treated teeth with higher sensitivity and accuracy [21,22,23].

The purpose of this study was to investigate the characteristics and the detection ability of vertical root fractures in endodontically treated teeth by intraoral radiography and CBCT.

## 2. Materials and Methods

We reviewed CBCT images of 50 patients (18 males and 32 females; age range of 16–86 years; mean age 58.7 ± 16.0 years) with confirmed root fractures in endodontically treated teeth after extraction. They were examined for root fractures by CBCT in our university hospital from January 2017 to August 2019. Ten nonendodontically treated teeth were also examined at this time; however, they were excluded from this study as we were concerned with analyzing root fractures in endodontically treated teeth. The root fractures of all patients were confirmed after the extraction of tooth. All patients received intraoral radiography before CBCT for the diagnosis of root fracture, which was doubtful from the clinical observation but not certain without radiography. The intraoral radiography and CBCT images were independently evaluated by two radiologists, and any discrepancies were resolved by consensus. This study was approved by the Ethics Committee of the Nippon Dental University School of Life Dentistry at Niigata (ECNG-R-318).

Intraoral radiographs were obtained by an intraoral machine (HELIODENT Plus; Sirona Dental Systems, Tokyo, Japan) with a dental protocol: tube current, 7 mA; tube voltage, 70 kV.

CBCT image was acquired using a CBCT unit (Fine Cube; Yoshida, Tokyo, Japan) using the CBCT parameters with the standard mode: tube current, 4.00 mA; tube voltage, 90.00 kV. The field of view was 81 mm × 81 mm, and the rotation time was set at 16.8 s. The protocol was set to the thickness of 0.144 mm, and the axial, cross-sectional, and parasagittal multiplanar reformation (MPR) images and 3D images were obtained [24].

Fracture directions of the 50 root fractures in endodontically treated teeth were investigated at first, and we then divided these into vertical and horizontal root fractures. Of the 50 root fractures in endodontically treated teeth, 36 root fractures were vertical root fractures, and these were the fractures used for this study. Characteristics of 36 vertical root fractures in endodontically treated teeth were investigated. Cause of fracture was divided into traumatized teeth and nontraumatized teeth according to trauma history, and the numbers of each tooth were counted. Most of the coronal restorations were followed by crown regardless of the kind of core construction. Core construction was investigated: the number of metal cores, composite resin cores, and absence of cores were counted. Concerning the kind of teeth, the number of vertical root fractures at the anterior, premolar, and molar teeth were counted. Fracture direction in the vertical root fracture was divided into bucco-lingual and mesio-distal fractures, and each tooth number was counted. Bucco-lingual and mesio-distal fractures in 36 vertical root fractures were further investigated concerning the position of teeth (upper and lower teeth) and the kind of teeth (anterior, premolar, and molar teeth).

Detection ability of vertical root fractures in endodontically treated teeth by intraoral radiography and CBCT was examined by counting the number of detectable and undetectable vertical root-fractured teeth, including both bucco-lingual and mesio-distal fractures. The detection ability was decided according to the consensus of two radiologists at the time that each radiograph was taken.

Statistical analyses for the characteristics of vertical root fractures and characteristics of bucco-lingual and mesio-distal fractures were performed by χ^2^ test in the case that expected number was larger than five. Detection ability for the vertical root fractures using intraoral radiography and CBCT were analyzed by cross-tabulation. Regarding the cross-tabulation, Yates’s correction was used for the continuity correction. When there were statistically significant cross-tabulations, residual analysis was performed. Statistical analysis software (SPSS 17.0, SPSS JAPAN, Tokyo, Japan) was used for the statistical analysis, and differences of α < 0.05 were considered significant.

## 3. Results

Table 1 shows the fracture direction of the 50 root fractures in endodontically treated teeth. The number of vertical root fractures was larger than that of horizontal root fractures (χ^2^ (1) = 9.68, *p* = 0.002).

**Figure 1:**
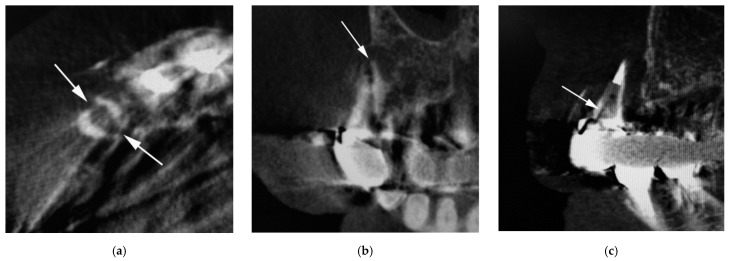
Cone-beam computed tomography (CBCT) image of vertical root fractures in endodontically treated teeth (bucco-lingual fracture). (**a**) Axial image; (**b**) cross-sectional multiplanar reformation (MPR) image; and (**c**) parasagittal MPR image. Arrow indicates part of root fracture.

**Figure 2:**
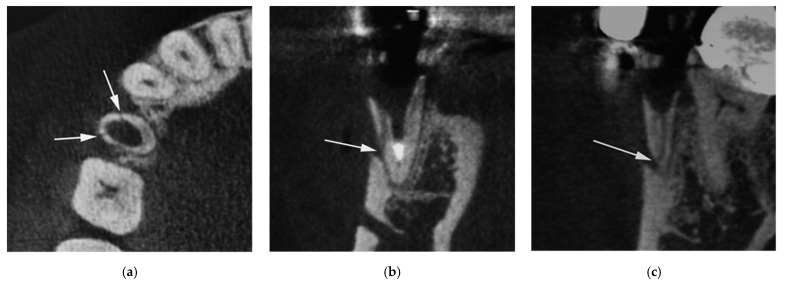
CBCT image of vertical root fractures in endodontically treated teeth (mesio-distal fracture). (**a**) Axial image; (**b**) cross-sectional MPR image; (**c**) parasagittal MPR image. Arrow indicates part of root fracture.

Characteristics of 36 vertical root fractures are shown in Table 2. All the vertically fractured teeth were nontraumatized teeth. The occurrence of vertical root fracture was not different among metal core, composite resin core, and no core (χ^2^ (2) = 1.17, *p* = 0.558). The number of vertical root-fractured teeth differed significantly among the anterior, premolar, and molar teeth (χ^2^ (2) = 10.50, *p* = 0.005), and the number of vertical root fractures in premolar teeth was largest. The number of vertical root fractures was significantly different between the bucco-lingual fracture (Figure 1) and mesio-distal fracture (Figure 2) (χ^2^ (1) = 4.00, *p* = 0.046), and the number of the bucco-lingual fractures was twice that of the mesio-distal fractures.

Bucco-lingual and mesio-distal fractures in 36 vertical root fractures were further investigated for the position of teeth (upper and lower teeth) and the kind of teeth (anterior, premolar, and molar teeth) (Table 3). The number of vertical root fractures was not significantly different between the bucco-lingual and mesio-distal fractures, both in the upper (χ^2^ (1) = 0.89, *p* = 0.346) and lower teeth (χ^2^ (1) = 3.56, *p* = 0.059). There were statistically significant differences between the bucco-lingual and mesio-distal fractures in the premolar teeth (χ^2^ (1) = 5.76, *p* = 0.016), and the number of bucco-lingual fractures was larger than that of the mesio-distal fractures. 

The results of the abilities of intraoral radiography and CBCT in the detection of vertical root fractures were shown in Table 4. Concerning the bucco-lingual fracture, the detection ability was statistically significantly different between intraoral radiography and CBCT (χ^2^ (2) = 19.29, *p* < 0.001, V = 0.68). The residual analysis results illustrated that bucco-lingual fractures were detectable using CBCT but were undetectable by intraoral radiography (*p* < 0.05). Regarding the mesio-distal fractures, the detection ability was also statistically significantly different using radiography (χ^2^ (2) = 20.17, *p* < 0.001, V = 1.00). The residual analysis results showed that mesio-distal fractures were detectable using CBCT but were undetectable by intraoral radiography (*p* < 0.05).

## 4. Discussion

Root fracture is an increasingly more prevalent cause of tooth loss than that of caries or periodontal disease [1]. In this study, the characteristics of vertical root fractures in endodontically treated teeth were investigated, and the detection abilities of intraoral radiography and CBCT were examined. The results showed the adequate radiography for the diagnosis of vertical root fractures in endodontically treated teeth.

The CBCT parameters at our hospital were as follows: standard mode (tube voltage, 90.0 kV; tube current, 4.00 mA; rotation time, 16.8 s; field of view, 81 mm × 81 mm; thickness, 0.144 mm), high-density mode (tube voltage, 90.0 kV; tube current, 4.00 mA; rotation time, 33.5 s; field of view, 81 mm × 81 mm; thickness, 0.144 mm), and high-resolution mode (tube voltage, 90.0 kV; tube current, 4.00 mA; rotation time, 16.8 s; field of view, 56 mm × 56 mm; thickness, 0.099 mm) [25]. High-resolution mode was sharper than both standard mode and high-density mode for the analysis of surgical specimens in segmental mandibulectomy [26]. However, standard mode was used for patients as the oral and maxillofacial protocols at our hospital because evaluation of another dentomaxillofacial lesion was necessary for diagnosis and treatment planning in dental practice [24].

Fracture directions of the 50 root fractures in endodontically treated teeth were investigated, and it was shown that 72% of the root fractures were vertical and 28% were horizontal. Therefore, this study put the vertical root fracture in endodontically treated teeth into focus. One report indicated that vertical root fracture was the third most common cause of extraction in endodontically treated teeth [27]. In endodontically treated teeth, dentin exhibits more plastic deformation than that of nonendodontically treated teeth, and the dehydration of dentin increases stiffness [28]. Furthermore, excessive removal of dentin during coronal enlargement [29] might lead to root fracture. Number of vertical root fractures is reported to be larger than that of horizontal root fractures [7], and our results supported this report. When teeth are endodontically treated, a spreader is added to create a wedging effect during lateral condensation, and this can cause vertical root fracture [30,31].

Characteristics of 36 vertical root fractures were investigated. All the vertically fractured teeth in this study were nontraumatized teeth. It is reported that vertical root fracture is larger in nontraumatized teeth, and traumatized teeth tend to have horizontal root fractures [32]. The result of this study supported the previous report [32]. Concerning the core construction, the numbers of metal cores and those with no core were larger compared to that of the composite resin cores in this study. The occurrence of vertical root fracture did not differ among the core construction. This result was inconsistent with previous studies that found that metal cores easily cause root fracture compared to that of cores with lower modulus of elasticity, such as resin core or fiber post [33,34]; conversely, the result supported a report that showed there were not significant differences in root fracture incidence between metal core and fiber post [35]. Regarding limitations of this study, the core construction did not influence the occurrence of vertical root fracture; however, the influence of core construction on vertical root fracture should be investigated owing to the increasing number of fractured teeth. 

Vertical root fractures occurred easily in premolar teeth, and the number was larger in bucco-lingual fractures compared to that of mesio-distal fractures. Some reports mentioned that the frequency of vertical root fractures was larger in premolars [36,37]. The anatomy of the premolar teeth is flat, and its roots are thin, with a smaller mesio-distal diameter; namely, an oval diameter in bucco-lingual direction [7]. Therefore, vertical root fracture would easily occur in the premolar teeth, and the fracture direction tended to be bucco-lingual in our study.

Bucco-lingual and mesio-distal fractures in 36 vertical root fractures were further examined regarding the position and type of teeth. The results showed that the number of bucco-lingual and mesio-distal fractures did not differ in either upper or lower teeth. However, the number of bucco-lingual fractures (76.2%) was larger than that of the mesio-distal fractures (23.8%) in premolar teeth. The numbers of bucco-lingual and mesio-distal fractures were not different in the anterior or molar teeth. This result suggested that vertical root fracture was more common in premolar teeth, and most fractures occurred in a bucco-lingual direction. The anatomy of the premolar teeth with smaller mesio-distal diameter and an oval diameter in a bucco-lingual direction may cause the bucco-lingual fracture [7]. Additionally, canal preparation was performed roundly in the oval canal, and the remaining dentin thickness decreased; therefore, the susceptibility to fracture increased [30].

The detection ability of vertical root fracture was greater in CBCT compared to that of intraoral radiography, both in the bucco-lingual and mesio-distal fractures. Most of the vertical root fractures were difficult to diagnose via intraoral radiography; therefore, CBCT is needed for the diagnosis of vertical root fractures. Concerning the mesio-distal fracture, all the vertical root fractures could not be diagnosed by intraoral radiography. On the other hand, 25% of the bucco-lingual fractures were detectable by intraoral radiography. This result suggested that if the fracture direction of the bucco-lingual fracture was parallel to the propagation direction of the radiation, the bucco-lingual fracture could be detected by intraoral radiography. Therefore, we recommend setting the propagation direction of the radiation parallel to the direction of the bucco-lingual vertical fracture if possible. Two cases of bucco-lingual vertical fractures could not be diagnosed by CBCT because of metal artifacts. A limitation of CBCT is with metal artifacts because they make examination results difficult to interpret [38]. The bucco-lingual fracture was detectable in 91.7% of fractures, and mesio-distal fracture was detectable in 100% of the fractures by CBCT in this study. These results were similar to that of previous reports that mentioned that CBCT can be used successfully for the detection of vertical root fracture, both in endodontically and nonendodontically treated teeth [21,22,23]. However, it was reported that visualization is not always possible in root-filled teeth via CBCT imaging [39]. In this study, it was sometimes impossible to diagnose vertical root fracture using CBCT because of the interference of metal artifacts. Therefore, it would be recommended to remove the metal before conducting radiography using CBCT. The results of this study suggest that CBCT is an adequate radiographic method for the diagnosis of vertical root fracture, both in bucco-lingual and mesio-distal fractures. 

The limitation of this study was that the sample number of root-fractured teeth was small. In future studies, a greater number of teeth should be examined to clarify the characteristics of vertical root fractures.

## 5. Conclusions

This study investigated the characteristics of vertical root fractures in endodontically treated teeth and the detection abilities of intraoral radiography and CBCT. The results made it clear that vertical root fracture occurs easily in premolar teeth with a bucco-lingual direction, and they also suggest that CBCT is an adequate radiographic method for the diagnosis of vertical root fractures in both bucco-lingual and mesio-distal fractures.

## Figures and Tables

**Table 1:** Fracture direction of 50 root fractures in endodontically treated teeth.

Parameters	Number of Root Fractures	*p* Value
Fracture direction	50 (100%)	0.002
Vertical fracture	36 (72.0%)	
Horizontal fracture	14 (28.0%)	

**Table 2:** Characteristics of 36 vertical root fractures.

Parameters	Number of Root Fractures	*p* Value
Cause of fracture	36 (100%)	-
Traumatized teeth	0 (0%)	
Nontraumatized teeth	36 (100%)	
Core construction	36 (100%)	0.558
Metal core	14 (38.9%)	
Composite resin core	9 (25.0%)	
No core	13 (36.1%)	
Kind of teeth	36 (100%)	0.005
Anterior teeth	9 (25.0%)	
Premolar teeth	21 (58.3%)	
Molar teeth	6 (16.7%)	
Fracture direction	36 (100%)	0.046
Bucco-lingual fracture	24 (66.7%)	
Mesio-distal fracture	12 (33.3%)	

**Table 3:** Bucco-lingual and mesio-distal fractures in 36 vertical root fractures.

Parameters	Number of Bucco-Lingual Fractures	Number of Mesio-Distal Fractures	*p* Value
Position of teeth			
Upper teeth	11 (61.1%)	7 (38.9%)	0.346
Lower teeth	13 (72.2%)	5 (27.8%)	0.059
Kind of teeth			
Anterior teeth	5 (55.6%)	4 (44.4%)	-
Premolar teeth	16 ^a^ (76.2%)	5 ^b^ (23.8%)	0.016
Molar teeth	3 (50.0%)	3 (50.0%)	-

Note: Significant difference was found between ^a^ and ^b^ (*p* < 0.05).

**Table 4:** Detection ability of vertical root fractures.

Detection Ability	Intraoral Radiography	CBCT
Bucco-lingual fracture		
Detectable	6 (25.0%) ^a^	22 (91.7%) ^b^
Undetectable	18 (75.0%) ^c^	2 (8.3 %) ^d^
Mesio-distal fracture		
Detectable	0(0%) ^e^	12 (100%) ^f^
Undetectable	12 (100%) ^g^	0 (0%) ^h^

Note: Significant differences were found between ^a^ and ^b^, ^c^ and ^d^, ^e^ and ^f^, and ^g^ and ^h^ (*p* < 0.05).

## Data Availability

The data presented in this study are available on request from the corresponding author. The data are not publicly available due to the authors’ work.

## References

[1] Liao W.C., Chen C.H., Pan Y.H., Chang M.C., Jeng J.H. (2021). Vertical root fracture in non-endodontically and endodontically treated teeth: Current understanding and future challenge. J. Pers. Med..

[2] Silva L.R., de Lima K.L., Santos A.A., Leles C.R., Estrela C., de Freitas Silva B.S., Yamamoto-Silva F.P. (2021). Dentin thickness as a risk factor for vertical root fracture in endodontically treated teeth: A case-control study. Clin. Oral Investig..

[3] Valenti-Obino F., Di Nardo D., Quero L., Miccoli G., Gambarini G., Testarelli L., Galli M. (2019). Symmetry of root and root canal morphology of mandibular incisors: A cone-beam computed tomography study in vivo. J. Clin. Exp. Dent..

[4] Ok E., Altunsoy M., Nur B.G., Aglarci O.S., Çolak M., Güngör E. (2014). A cone-beam computed tomography study of root canal morphology of maxillary and mandibular premolars in a Turkish population. Acta Odontol. Scand..

[5] Zhang R., Yang H., Yu X., Wang H., Hu T., Dummer P.M. (2011). Use of CBCT to identify the morphology of maxillary permanent molar teeth in a Chinese subpopulation. Int. Endod. J..

[6] de Pablo O.V., Estevez R., Heilborn C., Cohenca N. (2012). Root anatomy and canal configuration of the permanent mandibular first molar: Clinical implications and recommendations. Quintessence Int..

[7] García-Guerrero C., Parra-Junco C., Quijano-Guauque S., Molano N., Pineda G.A., Marín-Zuluaga D.J. (2018). Vertical root fractures in endodontically-treated teeth: A retrospective analysis of possible risk factors. J. Investig. Clin. Dent..

[8] Yoshino K., Ito K., Kuroda M., Sugihara N. (2015). Prevalence of vertical root fracture as the reason for tooth extraction in dental clinics. Clin. Oral Investig..

[9] Khandelwal A., Palanivelu A. (2020). Decision analysis for management of vertical root fracture. JEMDS.

[10] Yoshino K., Ito K., Kuroda M., Sugihara N. (2018). Duration from initial symptoms to diagnosis of vertical root fracture in dental offices. Bull. Tokyo Dent. Coll.

[11] Tsesis I., Kamburoğlu K., Katz A., Tamse A., Kaffe I., Kfir A. (2008). Comparison of digital with conventional radiography in detection of vertical root fractures in endodontically treated maxillary premolars: An ex vivo study. Oral Surg. Oral Med. Oral Pathol. Oral Radiol. Endodontol..

[12] Patel S., Dawood A., Whaites E., Pitt F.T. (2009). New dimensions in endodontic imaging: Part 1. Conventional and alternative radiographic systems. Int. Endod. J..

[13] Talwar S., Utneja S., Nawal R.R., Kaushik A., Srivastava D., Oberoy S.S. (2016). Role of cone-beam computed tomography in diagnosis of vertical root fractures: A systematic review and meta-analysis. J. Endod..

[14] Dias D.R., Iwaki L.C.V., de Oliveira A.C.A., Martinhão F.S., Rossi R.M., Araújo M.G., Hayacibara R.M. (2020). Accuracy of high-resolution small-volume cone-beam computed tomography in the diagnosis of vertical root fracture: An in vivo analysis. J. Endod..

[15] PradeepKumar A.R., Shemesh H., Nivedhitha M.S., Hashir M.M.J., Arockiam S., Uma Maheswari T.N., Natanasabapathy V. (2021). Diagnosis of vertical root fractures by cone-beam computed tomography in root-filled teeth with confirmation by direct visualization: A systematic review and meta-analysis. J. Endod..

[16] Melo S.L., Haiter-Neto F., Correa L.R., Scarfe W.C., Farman A.G. (2013). Comparative diagnostic yield of cone beam CT reconstruction using various software programs on the detection of vertical root fractures. Dentomaxillofac. Radiol..

[17] Jakobson S.J., Westphalen V.P., Silva N.U.X., Fariniuk L.F., Schroeder A.G., Carneiro E. (2014). The influence of metallic posts in the detection of vertical root fractures using different imaging examinations. Dentomaxillofac. Radiol..

[18] Saati S., Eskandarloo A., Falahi A., Tapak L., Hekmat B. (2019). Evaluation of the efficacy of the metal artifact reduction algorithm in the detection of a vertical root fracture in endodontically treated teeth in cone-beam computed tomography images: An in vitro study. Dent. Med. Probl..

[19] Moudi E., Haghanifar S., Madani Z., Alhavaz A., Bijani A., Bagheri M. (2014). Assessment of vertical root fracture using cone-beam computed tomography. Imaging Sci. Dent..

[20] Kobayashi-Velasco S., Salineiro F.C., Gialain I.O., Cavalcanti M.G. (2017). Diagnosis of alveolar and root fractures: An in vitro study comparing CBCT imaging with periapical radiographs. J. Appl. Oral Sci..

[21] Doğan M.S., Callea M., Kusdhany L.S., Aras A., Maharani D.A., Mandasari M., Adiatman M., Yavuz I. (2018). The evaluation of root fracture with cone beam computed tomography (CBCT): An epidemiological study. J. Clin. Exp. Dent..

[22] Valizadeh S., Khosaravi M., Azizi Z. (2011). Diagnostic accuracy of conventional, digital and cone beam computed tomography in vertical root fracture detection. Iran. Endod. J..

[23] Edlund M., Nair M.K., Nair U.P. (2011). Detection of vertical root fracture using cone beam computed tomography: A clinical study. J. Endod..

[24] Sue M., Oda T., Sasaki Y., Ogura I. (2018). Age-related changes in the pulp chamber of maxillary and mandibular molars on cone-beam computed tomography images. Oral Radiol..

[25] Ogura I., Ono J., Okada Y. (2018). Use of cone-beam computed tomography for evaluation of surgical specimen of medication-related osteonecrosis of the jaw. J. Oral Maxillofac. Radiol..

[26] Ogura I., Minami Y., Ono J., Kanri Y., Okada Y., Igarashi K., Haga-Tsujimura M., Nakahara K., Kobayashi E. (2021). CBCT imaging and histopathological characteristics of osteoradionecrosis and medication-related osteonecrosis of the jaw. Imaging Sci. Dent..

[27] Toure B., Faye B., Kane A.W., Lo C.M., Niang B., Boucher Y. (2011). Analysis of reasons for extraction of endodontically treated teeth: A prospective study. J. Endod..

[28] Soares C.J., Santana F.R., Silva N.R., Preira J.C., Pereira C.A. (2007). Influence of the endodontic treatment on mechanical properties of root dentin. J. Endod..

[29] Abdo S.B., Darrat A.A., Masaudi S.M., Luddin N., Husein A., Khamis M.F. (2012). Comparison of over flared root canals of mandibular premolars filled with MTA and resin based material: An in vitro study. Smile Dent. J..

[30] Barreto M.S., Moraes Rdo A., Rosa R.A., Moreira C.H., Só M.V., Bier C.A. (2012). Vertical root fractures and dentin defects: Effects of root canal preparation, filling, and mechanical cycling. J. Endod..

[31] Uzunoglu E., Aktemur S., Uyanik M.O., Durmaz V., Nagas E. (2012). Effect of ethylenediaminetetraacetic acid on root fracture with respect to concentration at different time exposures. J. Endod..

[32] Malhotra N., Kundabala M., Acharaya S. (2011). A review of root fractures: Diagnosis, treatment and prognosis. Dent. Update.

[33] Hikasa T., Matsuka Y., Mine A., Minakuchi H., Hara E.S., Van Meerbeek B., Yatani H., Kuboki T. (2010). A 15-year clinical comparative study of the cumulative survival rate of cast metal core and resin core restorations luted with adhesive resin cement. Int. J. Prosthodont..

[34] Fokkinga W.A., Kreulen C.M., Vallittu P.K., Creugers N.H. (2004). A structured analysis of in vitro failure loads and failure modes of fiber, metal, and ceramic post-and-core systems. Int. J. Prosthodont..

[35] Figueiredo F.E., Martins-Filho P.R., Faria-E-Silva A.L. (2015). Do metal post-retained restorations result in more root fractures than fiber post-retained restorations? A systematic review and meta-analysis. J. Endod..

[36] Safi Y., Aghdasi M.M., Ezoddini-Ardakani F., Beiraghi S., Vasegh Z. (2015). Effect of metal artifacts on detection of vertical root fractures using two cone beam computed tomography systems. Iran. Endod. J..

[37] Ardakani F.E., Razavi S.H., Tabrizizadeh M. (2015). Diagnostic value of cone-beam computed tomography and periapical radiography in detection of vertical root fracture. Iran. Endod. J..

[38] Junqueira R.B., Verner F.S., Campos C.N., Devito K.L., do Carmo A.M. (2013). Detection of vertical root fractures in the presence of intracanal metallic post: A comparison between periapical radiography and cone-beam computed tomography. J. Endod..

[39] Zhang L., Wang T., Cao Y., Wang C., Tan B., Tang X., Tan R., Lin Z. (2019). In vivo detection of subtle vertical root fracture in endodontically treated teeth by cone-beam computed tomography. J. Endod..

